# The regulatory effect and mechanism of traditional Chinese medicine on the renal inflammatory signal transduction pathways in diabetic kidney disease: A review

**DOI:** 10.1097/MD.0000000000039746

**Published:** 2024-09-20

**Authors:** Xiaoxia Li, Guoping Ma, Jin Liu, Guoqiang Zhang, Kexin Ma, Baozhu Ding, Wenjie Liang, Weifang Gao

**Affiliations:** a College of Integrated Traditional Chinese and Western Medicine, Hebei University of Chinese Medicine, Shijiazhuang, Hebei, China; b The First Hospital, Hebei Medical University, Shijiazhuang, Hebei, China; c Shijiazhuang Yiling Pharmaceutical Co., Ltd, Shijiazhuang, Hebei, China; d Rural Physician College, Hebei Medical University, Shijiazhuang, Hebei, China; e Hebei Key Laboratory of Integrative Medicine of Liver-Kidney Patterns, Institute of Integrative Medicine, Hebei University of Chinese Medicine, Shijiazhuang, Hebei, China.

**Keywords:** diabetic kidney disease, inflammatory injury, mechanism, signaling pathway, traditional Chinese medicine

## Abstract

Inflammatory injury is a critical factor in the occurrence and development of diabetic kidney disease (DKD). Signal transduction pathways such as the nuclear factor kappa beta (NF-κB), mitogen-activated protein kinase (MAPK), NOD-like receptor protein 3, and Smads are important mechanisms of inflammatory kidney injury in DKD, and the NF-κB pathway plays a key role. The inflammatory factor network formed after activation of the NF-κB pathway connects different signaling pathways and exacerbates renal inflammatory damage. Many traditional Chinese medicine compounds, single agents, effective components and active ingredients can regulate the expression of key molecules in the signaling pathways associated with inflammatory injury, such as transforming growth factor-β activated kinase 1, NF-κB, p38MAPK, NOD-like receptor protein 3, and Smad7. These treatments have the characteristics of multiple targets and have multiple and overlapping effects, which can treat DKD kidney inflammation and injury through multiple mechanisms and apply the “holistic concept” of traditional Chinese medicine.

## 1. Introduction

Diabetes is an inflammatory disease that seriously affects human health. Statistically, the incidence and mortality of diabetes are increasing at an alarming rate, and it is estimated that by 2030, the number of diabetic patients worldwide will reach 439 million.^[1]^ A persistent high-glucose environment is a risk factor for complications.^[2]^ Diabetic kidney disease (DKD) is the most common and serious complication of diabetes, and inflammatory damage is a key factor in the development of DKD.^[3]^ In DKD kidneys, inflammatory cells such as macrophages, neutrophils, and T cells are activated and infiltrate, inflammatory mediators such as cytokines, adhesion molecules, and chemical molecules are excessively produced, and inflammation-related signal transduction pathways are activated, eventually leading to inflammatory injury.^[4]^ Inflammation-related signaling pathways mainly include nuclear factor kappa beta (NF-κB), MAPK, NOD-like receptor protein 3 (NLRP3), and Smad7. Studies have shown that traditional Chinese medicine compounds, single agents, effective components and active ingredients can antagonize inflammatory renal tissue injury in DKD by regulating related inflammatory signaling pathways, thereby delaying the progression of DKD.^[5]^

## 2. Traditional Chinese medicine-mediated regulation of the NF-κB signaling pathway in DKD kidneys

### 2.1. NF-κB signaling pathway

The NF-ĸB protein family consists of 5 dimers: RelA (p65), RelB, c-Rel, p50, and p52. NF-κB is regulated by inhibitor of NF-κB (IκB). IκB proteins include IκBα, IκBβ, and IκBε. IκB binds to the NF-κB dimer, which exists in the cytoplasm but cannot translocate to the nucleus to activate downstream gene expression.^[6]^ The NF-κB signaling pathway can be activated by pathogen-associated molecular patterns (PAMPs), damage-associated molecular patterns (DAMPs), cytokines, lymphokines, and stress signals. PAMPs and DAMPs bind to Toll-like receptors, while interleukin-1β (IL-1β), tumor necrosis factor-α (TNF-α), and interleukin-18 (IL-18), and other cytokines bind to the corresponding cytokine receptors and activate transforming growth factor-β activated kinase 1 (TAK1) through a series of signal transduction pathways. Activated TAK1 phosphorylates NF-κB to inhibit protein kinase (IKK); activated IKK further phosphorylates and degrades IκB, promotes NF-κB translocation into the nucleus, and regulates the gene expression of inflammatory-related cytokines such as IL-1β, TNF-α, monocyte chemoattractant protein-1 (MCP-1), and interleukin-6 (IL-6).^[4]^

### 2.2. Regulatory mechanism of traditional Chinese medicine

Activation of the NF-κB signaling pathway plays a pivotal role in renal inflammatory injury in DKD. In DKD, hyperglycemia, advanced glycation end products (AGEs), reactive oxygen species (ROS), and other DAMPs, IL-1β, TNF-α, and other inflammatory factors can activate renal NF-ĸB signaling, leading to an increase in the production of inflammatory factors and aggravation of inflammatory damage in DKD kidneys.^[7]^ Many traditional Chinese medicines or their extracts can downregulate the expression of Toll-like receptors and myeloid differentiation factor 88 (MyD88) in renal tissue and inhibit the phosphorylation of IKK and IκBα proteins, thereby inhibiting the translocation of NF-κB and antagonizing the inflammatory response in DKD kidneys.

#### 2.2.1. Downregulation of TLR4 expression

The TLR4-NF-κB pathway is one of the important mechanisms of NF-κB signal transduction. NF-κB is a downstream effector of TLR4. TLR4 belongs to the Toll-like receptor family and is a pattern recognition receptor. High glucose activates TLR4, which activates NF-κB through a series of signal transduction pathways, promotes the expression of inflammatory factor-related genes, increases the production of proinflammatory cytokines such as IL-1β, IL-6, and MCP-1, and aggravates renal inflammatory damage.^[8–10]^ Many active ingredients or effective components of traditional Chinese medicines can inhibit the TLR4-NF-κB pathway and antagonize the inflammatory damage in DKD kidneys. Berberine, the active component of the traditional Chinese medicine *Coptis chinensis*, lowers blood sugar and protects the kidneys. Zhu L et al reported that berberine could inhibit the expression of TLR4 in the kidneys of DKD rats and high glucose-induced podocytes and inhibit the TLR4-NF-κB pathway. Berberine reduced the production of inflammatory factors such as TNF-α, IL-6 and IL-18, thereby alleviating the renal inflammatory response and podocyte damage in DN rats.^[8]^ Oridonin A, the active component of *Rubescens*, has anti-inflammatory, antioxidant, and immunomodulatory properties. Li et al found that high glucose could upregulate TLR4 expression in HBZY-1 rat mesangial cells. Oridonin A downregulated the expression of TLR4 and inhibited the NF-κB pathway, which is an important anti-inflammatory mechanism in DKD.^[9]^ Angelica polysaccharide,^[10]^ the main active component of *Angelica sinensis*, triptolide,^[11]^ the active component of *Tripterygium wilfordii*, and stilbene glycoside, the active component of *Polygonum multiflorum*,^[12]^ also have similar effects.

#### 2.2.2. Decreased MyD88 expression

MyD88 is an important linker molecule between the TLR4-NF-κB signaling pathway and the LI-1R-NF-κB signaling pathway. IL-1β binding to IL-1R, DAMPs and TLR4 recruits MyD88, MyD88 transduces signals and activates downstream TAK1, and the TAK-IKK-IκB cascade leads to the entry of NF-κB into the nucleus and initiation of inflammatory gene transcription. Catalpol is the main active component of the traditional Chinese medicine *Rehmannia glutinosa*. Chen Y et al reported that catalpol could reduce the protein expression of MyD88 in mouse kidney podocytes induced by high glucose, interrupt the signaling of MyD88, and could not effectively activate NIK or phosphorylate IKK, which in turn failed to relieve the inhibitory effect of IκB on NF-κB and inhibit the nuclear transfer of NF-κB, thereby reducing the production of the downstream inflammatory factors IL-6, TNF-α, and IL-1β and improving DKD podocyte injury.^[13]^ 7-Hydroxycoumarin is a derivative of coumarin, the active component of *A sinensis* and other traditional Chinese medicines. 7-Hydroxycoumarin has strong anti-inflammatory effects. Wang HQ et al showed that 7-hydroxycoumarin could significantly downregulate the expression of MyD88 in the kidney tissue of DKD rats, thereby inhibiting the phosphorylation of IκBα and the release of proinflammatory cytokines and antagonizing renal inflammatory injury.^[14]^

#### 2.2.3. Inhibiting the phosphorylation of IKK and IκBα

IKK is an important kinase in the NF-κB signaling pathway, and IĸBα is an important inhibitory protein in the NF-κB signaling pathway. IĸBα binds to NF-κB and inhibits its translocation to the nucleus. Activated IKK can phosphorylate and degrade IκB, translocate NF-κB to the nucleus, activate the transcription of inflammatory factor genes, and increase the production of inflammatory factors. In addition, inflammatory factors can also activate NF-κB to further aggravate DKD.^[15]^ Some traditional Chinese medicine compounds or active components can inhibit the phosphorylation of IκBα, thereby inhibiting the entry of NF-ĸB into the nucleus. The main component of Huangkui capsule is the extract of the traditional Chinese medicine hollyhock flower, which can clear dampness and heat, detoxify and reduce tumescence and has been widely used in the clinical treatment of DKD. Han W et al reported that Huangkui capsules could downregulate the expression of IKK in the kidneys of DKD rats and inhibit the phosphorylation of IKK, thereby reducing the degradation of the inhibitory protein IκB, inhibiting the translocation of NF-κB, and antagonizing the inflammatory response in the DKD kidney.^[16]^ The Qi-dan-di-huang Decoction is composed of Astragalus, *Salvia miltiorrhiza*, *R glutinosa*, Chinese yam, and licorice. This treatment has the effects of nourishing qi, nourishing yin, promoting blood circulation and removing blood stasis. Ma et al showed that this recipe could reverse the phosphorylation of IκBα in the kidneys of DKD rats and downregulate P65 expression, thereby inhibiting the production and release of inflammatory cytokines and improving the physiological and biochemical indicators of DKD in rats and the inflammatory state in vivo.^[17]^ Calycosin is an effective component of the traditional Chinese medicine Astragali Radix, which has anti-inflammatory, antioxidant, and antiapoptotic effects. Zhang YY et al reported that calycosin could reduce the phosphorylation of IĸBα in mouse renal tubular epithelial cells, downregulate the expression of P65, and weaken renal inflammatory injury in DKD.^[18]^ Gentiopicroside is an active component of the traditional Chinese medicine gentian, which can lower blood lipids and exert anti-inflammatory and antioxidant effects. This drug can inhibit activation of the NF-κB pathway by inhibiting the degradation of IĸBα in rat glomerular mesangial cells induced by high glucose and improve renal injury and renal fibrosis progression caused by inflammatory factors.^[19]^ Berberine also has a similar effect.^[8]^

#### 2.2.4. Direct inhibition of p65 protein expression

p65, also known as RelA, is an important member of the NF-κB family. Some traditional Chinese medicine compounds or active components can directly inhibit NF-κB (p65) and exert anti-inflammatory effects. Huopu Xialing Decoction comes from the “Yi Yuan” written by Shi Shoutang in the Qing Dynasty. This treatment is composed of 11 traditional Chinese medicines (Huoxiang, Magnolia, Ginger Pinellia, Poria, Almond, Polyporus, Coix Seed, Bai Kou Ren, Dandougu, Alisma, and Tongcao), which have significant anti-inflammatory effects. Zhong Yanhua et al found that Huopu Xialing decoction could inhibit the protein expression of p65, IL-1β, and TNF-α in lipopolysaccharide-induced HBZY-1 rat mesangial cells and the kidneys of DKD rats and reduce the serum inflammatory factors TNF-α and IL-1β and IL-6, suggesting that Huopu Xialing decoction reduced kidney inflammation by inhibiting p65 activity.^[20]^ *Toona sinensis* is a dried and ripe fruit that has antioxidant, antiatherosclerosis/inflammatory, antidiabetic and other effects, and n-butanol extracts of *T sinensis*^[21]^ and Liuwei Dihuang Pill^[22]^ also have similar effects.

## 3. Traditional Chinese medicine-mediated regulation of the MAPK signaling pathway in DKD kidneys

### 3.1. MAPK signaling pathway

The MAPK family is a group of serine-threonine protein kinases that includes 3 subfamilies: extracellular regulated kinase 1/2, p38 MAPK and c-Jun N-terminal kinase (JNK). MAPK can be activated by MAPK kinase (MAPKK/MAP2K), and MAP2K includes the MKK and MEK families. MAP2K can be activated by MAP2K kinases (MAPKK kinase, MAPKKK/MAP3K), such as TAK1, and activated MAP3K can phosphorylate MAP2K, which in turn phosphorylates and activates MAPK. Activated MAPK is transferred to the nucleus, phosphorylates transcription factors such as NF-κB, indirectly or directly leads to the production of inflammatory cytokines such as IL-1β, TNF-α, and IL-6, and participates in the inflammatory response.^[23]^

### 3.2. Regulatory mechanism of traditional Chinese medicine

During the development of DKD, various factors, such as hyperglycemia, oxidative stress, and proinflammatory factors, can activate the MAPK signaling pathway, promote the activation of downstream inflammatory cells, and induce the release inflammatory mediators, leading to inflammatory damage to renal tissue.^[24]^ In an experimental study of DKD rats, it was found that high glucose could induce macrophages to differentiate into a proinflammatory phenotype, promote TAK1 activation, and then activate the TAK-MKK-p38MAPK and TAK-IKK-NF-κB pathways, inducing the release of inflammatory factors, such as TNF-α, IL-1β, and MCP-1, and thereby exacerbating renal pathological damage.^[25]^ Chen P et al reported that high glucose could upregulate p38MAPK pathway expression in HK-2 human proximal tubular epithelial cells, promote the production of inflammatory cytokines such as TNF-α, IL-6, and IL-1β, and aggravate the inflammatory response in HK-2 cells.^[26]^

#### 3.2.1. Inhibition of TAK1 expression

TAK1 belongs to the MAPKKK family, which is also known as mitogen-activated protein kinase 7 (MAP3K7), and this factor can be stimulated by transforming growth factor-β1 (TGF-β1), TNF-α, IL-1, TLR ligands, and hypoxia. TAK1 is involved in various pathophysiological processes, such as cell growth, inflammation, immune response, and oxidative metabolism.^[27]^ TAK1 is involved in the p38 MAPK inflammatory signaling pathway and is involved in NF-κB pathway activity mediated by TNFR, IL-1R, and TLR.^[28]^ Studies have shown that the TAK1-MAPK signaling cascade plays a key role in initiating DKD inflammatory injury. After mouse bone marrow-derived macrophages were induced by AGEs, the expression levels of P-TAK1 and TAK1 and the phosphorylation levels of their downstream factors p38 MAPK and JNK increased, and the activity of NF-κB increased. The mRNA levels of the inflammatory factors TNF-α and MCP-1 also increased accordingly.^[29]^ Guo Shuai et al reported that the traditional Chinese medicine for removing blood stasis and dredging collaterals composed of the Chinese herbs Chuanxiong, Danshen, Dilong, leech, and Quan scorpion could significantly reduce the protein expression of TAK1 and JNK in the kidney tissue of DKD rats and inhibit the infiltration and activation of renal macrophages and the release of the inflammatory factors MCP-1, IL-β, and TNF-α into the blood, suggesting that the traditional Chinese medicine used to remove blood stasis and dredging collaterals could reduce the renal inflammatory response by inhibiting the activation of the TAK1-JNK pathway.^[30]^

#### 3.2.2. Inhibition of MAPK phosphorylation

The phosphorylation of the TAK1-MKK-p38 MAPK cascade is an important mechanism associated with inflammatory injury in DKD kidneys. In DKD, MAPKKK-TAK1 is activated, which phosphorylates MAPKK-MKK3/6 and then phosphorylates p38MAPK, thereby activating the NF-κB signaling pathway and increasing the expression of inflammatory factors.^[31]^ Many traditional Chinese medicine compounds or their active components can inhibit the phosphorylation of p38MAPK and JNK to protect the kidney. Liuwei Dihuang Pill is composed of *R glutinosa*, *Cornus officinalis*, Chinese yam, peony, Poria, and Alisma, and Zhenwu Decoction is composed of Poria cocos, Peony, Atractylodes, ginger, and aconite. Xu et al reported that the phosphorylation level of p38 MAPK in the kidneys of DKD rats was higher than that in normal rats, and treatment with Liuwei Dihuang Pill and Zhenwu Decoction inhibited the phosphorylation of p38 MAPK in the kidneys of DKD rats, reduced renal inflammatory damage, and exerted renal protection.^[22]^ *T wilfordii* b, the active component of *T wilfordii*, also had this effect.^[31]^ Paeoniflorin is an active component of the traditional Chinese medicine *Paeonia lactiflora* and has anti-inflammatory, antioxidant and antiapoptotic properties. This drug can inhibit activation of the p38 MAPK and JNK pathways in rat pancreatic β cells treated with streptozotocin (STZ) to exert a protective effect on pancreatic β cells.^[32]^ Esculin is a derivative of coumarin, the active component of Chinese ash tree bark, and has antibacterial, anti-inflammatory and anti-allergic effects. Liu Y et al showed that esculin could inhibit STZ-induced p38 activity in DKD rat kidneys. MAPK and JNK phosphorylation inhibits the expression of these proteins and exerts an anti-inflammatory effect on DKD.^[33]^ The active components of ginseng, ginsenoside Rg5,^[34]^ rubescensine A,^[9]^ and catalpol,^[13]^ also have similar effects.

#### 3.2.3. Inhibition of inflammatory cytokines

Inflammatory factors are the products and activators of the MAPK signaling pathway and are the key links in the positive feedback associated with MAPK signaling. During DKD, activated MAPK translocates to the nucleus, phosphorylates NF-κB, and increases the expression of inflammatory factors such as TNF-α, IL-1β, IL-6, and IL-18. These factors further stimulate the p38MAPK signaling pathway, forming a positive feedback loop, aggravating the renal inflammatory response, and promoting the development of DKD.^[24]^

Many traditional Chinese medicines or their active components can inhibit the production of inflammatory factors by regulating the MAPK signaling pathway. Bekhogainsam decoction was created by Chang ChungChing in the Treatise on Febrile Disease (Shang Han Lun). It consists of gypsum, Anemarrhena, ginseng, japonica rice, and licorice. Bekhogainsam decoction is a classic prescription for “wasting-thirst” (Xiaoke syndrome in TCM) and for treating excess heat in the lungs and stomach and body and qi injuries. Meng X et al reported that this formula could inhibit the MAPK signaling pathway in DKD kidneys, reduce the production of IL-6, and antagonize renal inflammatory damage.^[35]^ Zishen Pill is composed of 3 herbs: Zhimu, Huangbai, and Cinnamon. It can nourish the kidney, clear away heat, and clear away Qi. Guo X et al showed that Zishen Pill could regulate the p38MAPK signaling pathway in the kidney in DKD and inhibit the production of IL-1β, IL-6, MCP-1, and other inflammatory factors, thereby reducing the renal inflammatory response.^[36]^ Baicalin is an active component of the traditional Chinese medicine *Scutellaria baicalensis*, which has antibacterial, anti-inflammatory, and antioxidant effects. Ma L et al reported that baicalin could inhibit the infiltration of inflammatory cells such as T lymphocytes, neutrophils, and macrophages by inhibiting the expression of various molecules associated with the MAPK signaling pathway in the kidneys of DKD mice. Furthermore, baicalin could reduce the expression of inflammatory factors such as IL-1β, IL-6, MCP-1, and TNF-α in renal tissue, improve the pathological state of renal tissue in DKD mice, and relieve renal inflammatory damage.^[37]^ Apigenin^[23]^ and esculin,^[33]^ the active components of the Chinese herbs Coriander and Selaginella, also have similar effects.

## 4. Traditional Chinese medicine-mediated regulation of the NLRP3 inflammasome signaling pathway in DKD kidneys

### 4.1. NLRP3 inflammasome signaling pathway

The inflammasome, also known as the pyrosome,^[38]^ is a protein complex formed by multiple proteins, such as pattern recognition receptors, in cells. The NLRP3 inflammasome is a multiprotein complex with a relative molecular mass of approximately 700,000 that is composed of NLRP3 receptor molecule, apoptosis-associated speck-like protein containing a CARD (ASC), and caspase-1.^[39]^ The NLRP3 inflammasome can be activated by a variety of PAMPs and DAMPs, thereby activating Caspase-1, which further cleaves IL-1β and IL-18 precursors into the inflammatory factors IL-1β and IL-18, respectively, while producing the pyroptotic effector protein Gasdermin D (GSDMD), which induces pyroptosis.^[40]^

### 4.2. Regulatory mechanism of traditional Chinese medicine

Activation of the NLRP3-Caspase-1-GSDMD signaling pathway is an important mechanism of DKD kidney inflammation.^[41,42]^ High glucose and high insulin increase AGEs, which leads to oxidative stress, promotes excessive ROS production, activates NLRP3, recruits ASC and procaspase-1, forms the NLRP3 inflammasome, and promotes procaspase-1 self-cleavage and activation. Furthermore, mature Caspase-1 promotes the cleavage and maturation of inflammatory factors such as IL-1β/IL-18 and cleaves GSDMD to generate N-terminal GSDMD to induce cell membrane perforation damage, leading to pyroptosis.^[43–45]^

#### 4.2.1. Reduced ROS production

ROS mainly refer to oxygen-containing compounds whose chemical properties are more active than oxygen. ROS are DAMPs and play an important role in accelerating inflammation. High glucose and high insulin can increase AGEs, lead to oxidative stress and excessive ROS generation, induce oligomerization and allostery in NLRP3, and activate the NLRP3 inflammasome.

Many traditional Chinese medicines or their active components can reduce ROS production and exert renal protection. NADPH oxidase 4 is a key redox enzyme in vivo and the main enzyme that catalyzes the production of ROS.^[46]^ Ginsenoside Rg5, the active component of ginseng, reduced NADPH oxidase 4 activity and inhibited ROS-mediated NLRP3 inflammasome activation after 5 weeks of high-fat diet feeding in STZ-induced DKD mice and reduced oxidative stress and the inflammatory response in DKD mice.^[34]^ Danggui Buxue Decoction is composed of Astragalus and Angelica, which can invigorate qi and generate blood and has similar effects.^[47]^ Dihydroquercetin is a flavonoid compound extracted from Huangqi leaves and other traditional Chinese medicines. This compound has antioxidant and anti-inflammatory effects. Dihydroquercetin can inhibit high glucose-induced ROS generation in HBZY-1 rat glomerular mesangial cells and HK-2 human proximal tubular epithelial cells and reduce NLRP3 inflammasome activity, effectively reducing oxidative stress and injury in renal cells.^[48]^ Ginsenoside compound K, the main component of the traditional Chinese medicine ginseng, has a similar effect.^[49]^

#### 4.2.2. Inhibition of p66Shc activity

The ShcA protein family is part of the Src Homology 2 Domain Containing family, and p66Shc is one of the 3 members of the ShcA protein family. p66Shc is an adaptor protein that mainly regulates the cellular oxidative stress response and life cycle signal transduction pathways. This protein can oxidize cytochrome C in mitochondria to generate a large amount of ROS, resulting in cellular oxidative damage. The traditional Chinese medicine zingiberensis (yellow ginger) has anti-inflammatory, antitumor, and antioxidant effects. It has been reported that turmeric can inhibit the protein and mRNA expression of p66Shc in the glomerular cells of DKD mice and reduce 8-hydroxy-2-deoxyguanosine levels. 8-Hydroxy-2-deoxyguanosine is an important biomarker of oxidative stress damage in DNA and inhibits the mRNA expression of NLRP3, Caspase-1 protein, and IL-1β in renal tissue.^[50]^ Therefore, zingiberensis can reduce oxidative stress and the inflammatory response and delay the development of DKD by inhibiting p66Shc activity and the NLRP3 inflammasome.

#### 4.2.3. Downregulated expression of inflammasome body components

NLRP3, ASC, and Caspase-1 are the receptor, linker and effector molecules of the NLRP3 inflammasome, respectively. Many traditional Chinese medicines or their active components can inhibit each molecule of the NLRP3 inflammasome to exert renal protection. It was reported that Huangkui capsule decreased the expression of NLRP3, Caspase-1, and IL-1β in the renal tubular epithelial cells and kidneys of DKD rats, inhibited activation of the NLRP3 inflammasome, and alleviated tubular epithelial-mesenchymal transition in DKD rats.^[16]^ The Chinese medicine sarsasapogenin,^[51]^ curcumin, the active component of the Chinese medicine turmeric, and ginsenoside compound K also have similar effects.^[49,52]^ *Cordyceps sinensis* is a fungus-caterpillar complex that is a traditional Chinese medicine for nourishing the lung and the kidney. This drug downregulated the protein and mRNA levels of NLRP3, ASC and Caspase-1 in the kidney tissue of DKD rats and high glucose-induced mouse podocytes and decreased the expression of downstream IL-1β and IL-18.^[53]^ Therefore, *Cordyceps sinensis* can reduce inflammatory injury in DKD and podocytes by inhibiting the activity of the NLRP3 inflammasome. The main component of saffron, crocin,^[54]^ and the natural flavonoid luteolin^[55]^ also have similar effects.

#### 4.2.4. Inhibition of inflammatory factors

NLRP3-Caspase-1-IL-1β/IL-18 is an important mechanism of DKD kidney inflammatory injury. Activation of the NLRP3 inflammasome leads to an increase in the production of IL-1β/IL-18, and IL-1β/IL-18 can bind to the corresponding cytokine receptors to initiate the NF-κB signaling pathway, providing the first signal to activate the NLRP3 inflammasome signaling pathway, forming a positive feedback loop and intensifying the inflammatory response. Many traditional Chinese medicines or their active components can reduce and inhibit IL-1β, IL-18, and other proinflammatory factors to inhibit NLRP3 signaling and exert renal protection. Wumei Pill is composed of black plum, asarum, ginger, coptis, angelica, aconite, prickly ash, cinnamon stick, ginseng, and phellodendron. This prescription can reduce IL-1β, IL-18, monocyte chemotactic protein-1α and the production of the macrophage-specific surface glycoprotein F4/80. This treatment also interferes with the expression levels of NLRP3, ASC, and Caspase-1 in pancreatic tissue,^[56]^ suggesting that Wumei Pill can reduce inflammatory injury and pyroptosis in pancreatic β cells by inhibiting the NLRP3 inflammasome. Artesunate is a derivative of artemisinin, the main component of the traditional Chinese medicine *Artemisia annua*, and has anti-inflammatory activity. Sun et al found that artesunate not only reduced the mRNA levels of IL-1β and IL-6 but also inhibited the expression of NLRP3 in cells.^[57]^ This result indicated that artesunate could alleviate renal inflammatory injury by inhibiting the NLRP3 inflammasome pathway and the production of inflammatory cytokines. Liquiritigenin and Astragalus polysaccharide IV are extracted from the traditional Chinese medicine licorice root and Astragalus, respectively, and have similar effects.^[58,59]^

## 5. Traditional Chinese medicine-mediated regulation of the Smad signaling pathway in DKD kidneys

### 5.1. Smads signaling pathway

The Smad signaling pathway is an important signal transduction pathway associated with organ fibrosis and is closely related to inflammatory injury. The members of the Smad protein family are all transcription factors that are the most important signal transduction molecules associated with TGF-β1. TGF-β1 first binds to TGF-β receptor, phosphorylates Smad2 and 3, and then recruits Smad4 to form a trimer that translocates to the nucleus, where it can act with other transcription factors on target genes, changing the expression of TGF-β1-responsive genes, which ultimately leads to fibrosis^[60,61]^; furthermore, this trimer can increase the transcription of the Smad7 promoter and upregulate Smad7 expression. Smad7 can competitively bind to TGF-β receptor, inhibit Smad2 and 3 phosphorylation, and play a regulatory role.

### 5.2. Regulatory mechanism of traditional Chinese medicine

The TGF-β1-Smad3 signaling pathway is activated in the kidneys of DKD patients, and the downregulation of Smad7 expression aggravated inflammatory injury. Studies have shown that renal Smad7 overexpression significantly inhibits DNA binding activity, nuclear translocation, the transcriptional activity of NF-κB (p65), and IL-1β and TNF-α-induced NF-κB-dependent inflammatory responses. Therefore, reasonable control of the level of Smad7 may be the key to the involvement of the TGF-β1/Smad3 and NF-κB signaling pathways in DKD kidney inflammation injury.^[62]^

#### 5.2.1. Inhibition of Smad7 expression

Smad7 is an inhibitory Smad and an adaptor protein. In DKD renal tissue associated with inflammation and fibrosis, the level of Smad7 is related to Smurf2. Smurf2 is the ubiquitin protein ligase of Smad7, which binds to Smad7 and promotes Smad7 degradation; once renal Smad7 is degraded, Smad3 is overactivated, and the TGF-β1/Smad3 signaling pathway is activated, which aggravates inflammatory injury and fibrosis in DKD kidney tissue.^[63–65]^ In addition, Smad7 can negatively regulate NF-κB signaling by inducing IκBα, preventing IκBα degradation, and inhibiting NF-κB signaling-driven inflammatory activation in vitro and in vivo.^[66]^

Tangshenfang can block activation of the NF-κB signaling pathway by inhibiting Smad7 and treat DKD kidney inflammation. According to reports, Tangshen Fang is composed of Astragalus, Gui Jianyu, *Citrus aurantium*, Rehmannia, Cornus, Rhubarb, and *Panax notoginseng*. The formula inhibited the expression of smurf2 and Smad3 in the kidney tissue of DKD rats, upregulated the expression of Smad7, improved the degradation of IκBα and the nuclear translocation of NF-κB (p65), reduced the protein and mRNA levels of TGF-β1, IL-1β, IL-6, and MCP-1, and effectively protected against renal fibrosis and inflammatory damage in DKD.^[67]^ Therefore, inhibiting smurf2-mediated Smad7 ubiquitin degradation may be the central mechanism by which Tangshenfang inhibits TGF-β1/smad3-mediated renal fibrosis and NF-κB-dependent renal inflammatory injury in DKD.

#### 5.2.2. Inhibition of TGF-β1 expression

TGF-β1 is most highly expressed in the kidney, affects the activity of proinflammatory cytokines, exerts chemotactic effects on T lymphocytes and neutrophils and is an important mediator of the development of kidney fibrosis and inflammation in DKD.^[58]^ Many traditional Chinese medicines or their active components can inhibit the inflammatory response and protect against kidney damage by regulating the TGF-β1/Smad signaling pathway. In a DKD rat model established by a high-fat, high-sugar diet and low-dose STZ injection, triptolide attenuated proteinuria and podocyte inflammatory injury by reducing the expression of TGF-β1, and reducing macrophage infiltration in the kidney.^[11]^ Li et al demonstrated that acetylshikonin, the main component of comfrey, could reduce the expression of IL-1β, IL-16, and MCP-1 in the kidney tissue of DKD mice, inhibit TGF-β1 expression and Smad2/3 phosphorylation, and upregulate Smad7 expression.^[68]^ Therefore, acetylshikonin alleviates renal fibrosis by inhibiting the TGF-β1/Smad pathway and the inflammatory response. Amygdalin is the main component of bitter almond, which has anti-inflammatory and antifibrotic properties and similar effects.^[69]^

## 6. Conclusion

**Figure 1.**
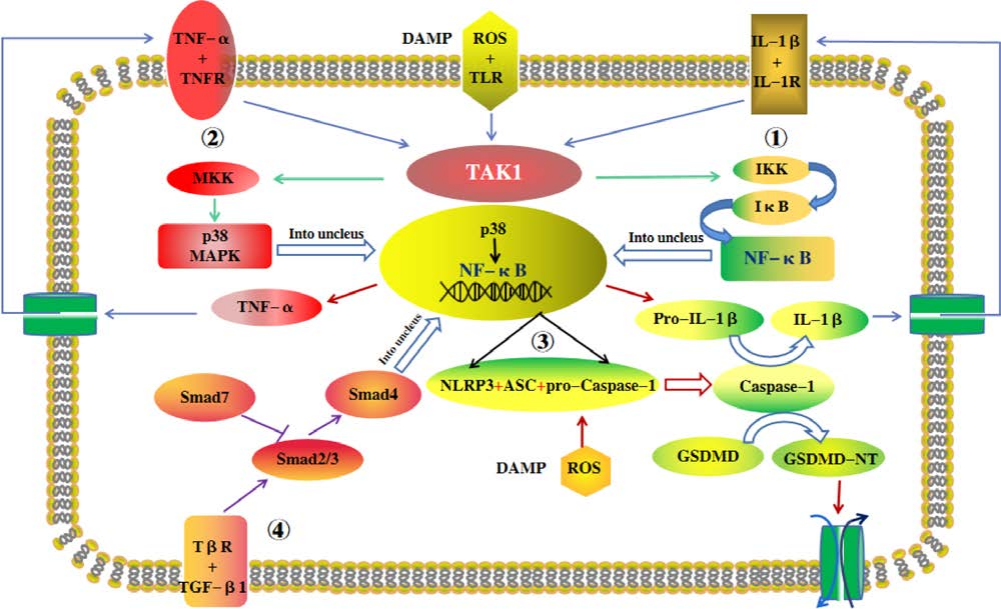
The inflammatory signal transduction pathways. ① NF-κB signaling pathway. During DKD, the excessively generated ROS binds to TLR4, which can sequentially activate the downstream TAK1 and IKK, resulting in the phosphorylation and degradation of IκB, and promoting the entry of NF-κB into the nucleus. After entering the nucleus, it regulates the gene expression of inflammatory factors such as IL-1β and TNF-α. ② MAPK signaling pathway. During DKD, ROS-TLR4 is activated, and it can also activate TAK1 and MKK3/6 in sequence, thereby activating p38MAPK protein molecules, the activated p38MAPK enters the nucleus, phosphorylates transcription factors such as NF-κB, promotes the expression of inflammatory factor genes, and generates Inflammatory factors such as IL-1β and TNF-α in turn activate TAK1, forming a positive feedback loop and aggravating renal inflammatory damage. ③ NLRP3 signaling pathway. Activation of NLRP3 pyroptotic bodies requires dual-signal stimulation. The first signal is provided by the activation of the NF-κB pathway. The second signal is provided by DAMPs such as cytoplasmic ROS, which makes NLRP3 undergo qualitative change and activate, recruiting ASC and pro-caspase-1 molecules to form pyroptotic bodies. The activated Caspase-1 can cleave the pro-IL-1β producedto form IL-1β by the first signal, and at the same time, the pyroptosis effector protein GSDMD-NT is produced, resulting in pyroptosis. ④ Smad4 signaling pathway. TGF-β1 first binds to TβR, phosphorylates Smad2 and 3, and then recruits Smad4 to form a trimeric translocation nucleus. Smad7 feedback inhibits the phosphorylation of Smad2 and 3, and inhibits the renal inflammatory response of NF-κB signaling pathway by negatively regulating NF-κB signaling. ASC = apoptosis-associated speck-like protein containing a CARD, DAMPs = damage-associated molecular patterns, DKD = diabetic kidney disease, GSDMD = Gasdermin D, IKK = inhibit protein kinase, IL-1β = interleukin-1β, MAPK = mitogen-activated protein kinase, NF-κB = nuclear factor kappa beta, NLRP3 = NOD-like receptor protein 3, ROS = reactive oxygen species, TAK1 = transforming growth factor-β activated kinase 1, TNF-α = tumor necrosis factor-α.

The inflammatory signal transduction pathways in the kidney associated with DKD include the NF-κB, MAPK, NLRP3, and Smad signaling pathways. The NF-κB signaling pathway is the core pathway associated with DKD kidney inflammatory injury. NF-κB translocates to the nucleus, directly regulates the expression of inflammatory factors and is closely related to other inflammatory signaling pathways. TAK1 kinase, which is an important upstream molecule of the MAPK signaling pathway, can activate IKK kinase, which is an upstream molecule of the NF-κB signaling pathway. After activation of the MAPK signaling pathway, activated MAPK translocates to the nucleus and phosphorylates NF-κB, increasing the production of inflammatory factors. The first signal for NLRP3 inflammasome activation is provided after the activation of the NF-κB signaling pathway. In the Smad signaling pathway, Smad7 inhibits NF-κB signaling pathway activation by negatively regulating NF-κB signaling, as shown in Figure 1.

The inflammatory factor network links different inflammatory signaling pathways in DKD kidneys, and inflammatory factors are key factors in the formation of a positive feedback loop. Inflammatory cytokines such as IL-1β can not only activate signaling pathways such as the NF-κB, MAPK, and NLRP3 inflammasomes but are also the products of these signaling pathways. TGF-β1 is not only the activating and product molecule of not only the TAK1-p38MAPK pathway but also the Smad pathway. Therefore, the formation of a positive feedback loop is an important reason for the aggravation of inflammatory injury. Furthermore, oxidative stress plays an important role in the activation of various inflammatory signaling pathways.

The regulatory effects of traditional Chinese medicines on renal inflammatory injury in DKD has multipoint, pleiotropic and overlapping characteristics. Multipoint means that a traditional Chinese medicine can act on multiple targets in a signaling pathway. For example, berberine can simultaneously inhibit the expression of TLR4, IκBα and P65 in the NF-κB signaling pathway. The pleiotropic effect means that a traditional Chinese medicine can interfere with different signaling pathways; for example, ginsenoside compound K and ginsenoside Rg5 can act on 3 different signaling pathways: NF-κB, MAPK, and NLRP3. Rubescensine A can act on 2 signaling pathways (NF-κB and MAPK) to antagonize inflammatory kidney injury in DKD. Overlapping means that different traditional Chinese medicines can act on the same pathway or the same target. For example, Tangshen recipe and acetyl shikonin can inhibit the expression of TGF-β1 and upregulate the Smad7 signaling pathway to achieve anti-inflammatory effects, as shown in Table 1. This multipathway and multitarget effect is suitable for the overall concept of traditional Chinese medicine, as well as the complexity of inflammatory signaling pathway activation in DKD kidneys. That is, these effects are mediated by multiple mechanisms. This paper only discussed the 4 signaling pathways closely related to inflammatory kidney injury in DKD. With the development of medical science and technology, new pathways will be discovered. Exploring the regulatory mechanism of traditional Chinese medicine on the signaling pathway associated with renal inflammatory injury in DKD is of great importance for the clinical treatment of DKD and the development of new drugs.

**Table 1:** Intervention effects of traditional Chinese medicine on inflammatory signaling pathways.

Inflammatory signaling pathway	Single-flavored Chinese medicine and compound	Active ingredients	Effective parts
NF-κB	^[16]^Huangkui Capsule ^[17]^Qi-dan-di-huang Decoction ^[20]^Huopu Xialing Decoction ^[22]^Liuwei Dihuang Pill	^[8]^Berberine ^[9]^Oridonin A Angelica^[10]^polysaccharide ^[11]^Triptolide ^[13]^Catalpol ^[18]^Caylcosin ^[19]^Gentiopicroside	^[12]^Stilbene glycoside ^[14]^7-Hydroxycoumarin ^[21]^N-butanol extract of *Toona sinensis*
MAPK	^[30]^The TCM for removing blood stasis and dredging collaterals ^[35]^Bekhogainsam Decoction ^[22]^Liuwei Dihuang Pill ^[22]^Zhenwu Decoction ^[36]^Zishen Pill	^[9]^Oridonin A ^[31]^*Tripterygium wilfordii* b ^[13]^Catalpol ^[34]^Ginsenoside Rg5 ^[32]^Paeoniflorin ^[37]^Baicalin	^[33]^Esculin ^[23]^Apigenin
NLRP3	^[51]^Sarsasapogenin ^[50]^Zingiberensis ^[53]^Cordyceps sinensis ^[47]^Danggui Buxue Decoction ^[56]^Wumei Pill ^[16]^Huangkui Capsule	^[34]^Ginsenoside Rg5 ^[49]^compound K ^[52]^Curcumin ^[54]^Crocin ^[58]^Liquiritigenin Astragalus^[59]^ polysaccharide IV	^[48]^Dihydroquercetin ^[55]^ Luteolin ^[57]^Artesunate
Smad (Smad7)	^[67]^Tangshen Fang	^[11]^Triptolide ^[68]^Acetylshikonin ^[69]^Amygdalin	

MAPK = mitogen-activated protein kinase, NF-κB = nuclear factor kappa beta, NLRP3 = NOD-like receptor protein 3.

## Acknowledgments

Thanks to all authors for their contributions to this manuscript and to the Foundation for its support.

## Author contributions

**Funding acquisition:** Wenjie Liang.

**Supervision:** Wenjie Liang.

**Writing – original draft:** Xiaoxia Li.

**Writing – review & editing:** Xiaoxia Li, Guoping Ma, Jin Liu, Guoqiang Zhang, Kexin Ma, Baozhu Ding, Wenjie Liang, Weifang Gao.
